# 

**Published:** 1853-02

**Authors:** 


### BOOKS RECEIVED.

*Tally's Materia Medica; or, Pharmacology and Therapeutics.*

THIS work was announced some months since, and the medical public has been looking with much curiosity for its appearance; and, judging from the numbers before us, this curiosity will be gratified. We have delayed to speak of the work, in order that we might get some idea of the plan, style, &c. That it will be original, the first numbers give unmistakeable evidence; but we very much fear that the profession will not generally endorse the propositions of the distinguished author. It is published in numbers of 64 pages each—Price 25 cents—by Jefferson Church, M.D., Springfield, Mass.

*Rankin's Abstract—Braithwaite's Retrospect.*

We have received these works from the publishers. They are filled, as usual, with interesting matter of both a scientific and practical character. The selections indicate the *hugeness* of Europe, and the *diminutiveness* of America.

We have received from the authors and publishers the following essays, pamphlets, &c.:—

Proceedings of the National Pharmaceutical Convention, held at Philadelphia, October, 1852.

History of the Medical Department of the University of Louisville, by Prof. L. P. Yandell.

Professional Reminiscences of Foreign Travel, by W. Channing, M.D., Boston.

Registration of Births, Deaths, and Marriages in Massachusetts, for the year ending 31st Dec., 1851, by J. Curtis, M.D., Boston.

Together with a number of Congressional and Legislative Documents.

### RECEIPTS

### FOR THE JOURNAL DURING THE MONTH OF JANUARY.

### Illinois.

| | |
|---|---|
| Dr. H. A. Raney, | 2 00 |
| " John Currie, | 1 00 |
| " Eyekiel Friend, | 2 00 |
| " G. H. Young, | 2 00 |
| " A. P. Finch, | 2 00 |
| " D. W. Stormont, | 2 00 |
| " John Warner, | 2 00 |
| " Boynton Tenney, | 2 00 |
| " R. C. Edgerton. | 2 00 |
| " Newell Sapp, | 2 00 |
| " J. H. Brown, | 2 00 |
| " B. A. Fuller, | 2 00 |
| " Jas. Woodward, | 2 00 |
| " Daniel Stahl, | 2 00 |
| " A. Hard, | 2 00 |
| " J. B. Glass, | 4 00 |
| " P. Dickinson, | 2 00 |
| " Thos. A. Brown | 2 00 |

### Ohio.

| | |
|---|---|
| Dr. A. H. Tyler, | 2 00 |

### Iowa.

| | |
|---|---|
| Dr. Ezra Van Fossen, | 2 00 |

### Indiana.

| | |
|---|---|
| Dr. Freeland, | 2 00 |
| " O. H. Martin, | 2 00 |
| " T. Newkirk. | 2 00 |
| " D. R. Crouse, | 4 00 |
| " A. Loftin, | 2 00 |
| " John A. Lloyd, | 2 00 |
| " A. M. Lewis, | 2 00 |
| " Wm. Mathews, | 2 00 |
| " S. Kelly, | 2 00 |

### Michigan.

| | |
|---|---|
| Dr. H. Knapp, | 2 00 |
| " P D. McArthur, | 1 00 |
| " J. D. Woodworth, | 2 00 |
| " R. D. Hawley, | 2 00 |

### Wisconsin.

| | |
|---|---|
| Dr. L B. W. Johnson | 2 00 |
| " R. W. Earle, | 2 00 |
| " J. R. Goodenough, | 2 00 |
| " Reuben Hall, | 2 00 |
| " Calvin D. Vilas, | 2 00 |
| " H. W. Hart, | 2 00 |
| " A. & E. N. Clark, | 2 00 |

### PROSPECTUS

### OF THE NEW SERIES OF THE

### NORTH-WESTERN MEDICAL AND SURGICAL JOURNAL.

EDITED BY

**W. B. HERRICK, M.D.,**

*Professor of Anatomy and Physiology in Rush Medical College; Surgeon to the U.S. Marine Hospital; and one of the Surgeons of the Ill. General Hospital.*

ASSISTED BY

**H. A. JOHNSON, M.D.**

THE NORTH-WESTERN MEDICAL AND SURGICAL JOURNAL will be published hereafter, on the first of each month, beginning in May, in numbers, each containing, exclusive of advertisements, Forty-eight pages, closely printed, with new type ordered expressly for this work; thus presenting at the end of the year a volume of nearly Six Hundred pages.

The change from a bi-monthly to a monthly Journal, has been made in accordance with the expressed wishes of numerous subscribers.

The editor, with his associate, Dr. JOHNSON, who will devote his time exclusively to the Journal, will endeavor, in addition to a full and complete Domestic Summary, to present numerous extracts from English, and translations from French and German Medical periodicals.

In addition to the Communications from Contributors, whose favors, it is hoped, will be continued, the Original Department will contain, from time to time, reports of Cases treated, and of Observations made, in the two Hospitals, now fully organized in Chicago.

### TERMS:

**TWO DOLLARS PER ANNUM, IN ADVANCE.**

As an inducement to Physicians and others to act as Agents, in extending the circulation of the Journal.

| | | | | |
|---|---|---|---|---|
| Three copies will be furnished to new subscribers for | | | | $5 00 |
| Five | " | " | " | 8 00 |
| Seven | " | " | " | 10 00 |

### TERMS OF ADVERTISING.

| | |
|---|---|
| One page, One year ............$20,00 | One page, single insertion........$5,00 |
| One-half page, One year, ...... 12,00 | One-half page, single insertion.... 3,00 |

☞ *Letters must be post-paid, and directed to the* "NORTH-WESTERN MEDICAL AND SURGICAL JOURNAL, *Chicago, Ill.*" *A list of receipts will appear in each No.*

